# Tissue Inhibitor of Matrix Metalloproteinases-1 Knockdown Suppresses the Proliferation of Human Adipose-Derived Stem Cells

**DOI:** 10.1155/2016/4761507

**Published:** 2016-04-27

**Authors:** Peihua Zhang, Jin Li, Yawei Qi, Xudong Tang, Jianfeng Duan, Li Liu, Zeyong Wu, Jie Liang, Jiangfeng Li, Xian Wang, Guofang Zeng, Hongwei Liu

**Affiliations:** ^1^Institute of Plastic Surgery, Stem Cell Research and Clinical Translation Center, Affiliated Hospital of Guangdong Medical University, Zhanjiang, Guangdong 524001, China; ^2^Institute of Biochemistry and Molecular Biology, Guangdong Medical University, Zhanjiang, Guangdong 524023, China; ^3^Department of Plastic Surgery, The First Affiliated Hospital of Jinan University, Key Laboratory for Regenerative Medicine, Ministry of Education, Guangzhou, Guangdong 510630, China

## Abstract

Tissue inhibitor of metalloproteinases-1 (TIMP-1) is a multifunctional matrix metalloproteinase, and it is involved in the regulation of cell proliferation and apoptosis in various cell types. However, little is known about the effect of TIMP-1 expression on the proliferation of adipose-derived stem cells (ADSCs). Therefore, TIMP-1 expression in the ADSCs was firstly detected by western blotting, and TIMP-1 gene was knocked down by lentivirus-mediated shRNA. Cell proliferation was then evaluated by MTT assay and Ki67 staining, respectively. Cell cycle progression was determined by flow cytometry. The changes of p51, p21, cyclin E, cyclin-dependent kinase 2 (CDK2), and P-CDK2 caused by TIMP-1 knockdown were detected by western blotting. The results indicated that ADSCs highly expressed TIMP-1 protein, and the knockdown of TIMP-1 inhibited cell proliferation and arrested cell cycle progression at G_1_ phase in the ADSCs possibly through the upregulation of p53, p21, and P-CDK2 protein levels and concurrent downregulation of cyclin E and CDK2 protein levels. These findings suggest that TIMP-1 works as a positive regulator of cell proliferation in ADSCs.

## 1. Introduction

Numerous studies have indicated that tissue inhibitors of metalloproteinases (TIMPs) play critical roles in the regulation of extracellular matrix (ECM) metabolism, tissue remodeling, and cellular behavior [1, 2]. It is widely well-recognized that TIMPs serve as the inhibitors of matrix metalloproteinases (MMPs) and other metalloproteinases and can regulate their activities and the proteinaceous ECM homeostasis. However, increasing evidence indicates that the range of TIMPs activities is more broader as they can also elicit metalloproteinase-independent biological activities [3, 4]. The human genome has four paralogous genes encoding endogenous inhibitors (TIMP-1, TIMP-2, TIMP-3, and TIMP-4) which all share several structural features. In this respect, TIMP-1 has been shown to be particularly interesting as it not only has a classical role as an inhibitor of MMPs but also has growth factor-like activity [5, 6]. Additionally, TIMP-1 has been shown to possess other activities in the regulation of various biological processes such as cell growth, differentiation, and apoptosis [7, 8].

Previous studies have demonstrated that human bone marrow-derived mesenchymal stem cells (MSCs) constitutively express high level of TIMP-1, and the silencing of TIMP-1 enhances cell migration [9]. Recent studies indicated that TIMP-1 ubiquitously is expressed in numerous human cells and tissues, and it is a cytoprotective released factor from embryonic stem (ES) cells, and its overexpression in ES cells attenuates adverse myocardial remodeling and improves cardiac function in the mice [10]. These findings prompted us to hypothesize that TIMP-1 expression may affect the basic functions of stem cells, such as cell proliferation and differentiation. Adipose-derived stem cells (ADSCs) are especially attractive multipotent progenitor cells because they are relatively abundant and more easily acquired. Additionally, ADSCs can differentiate into osteogenic, myogenic, chondrogenic, endothelial, adipogenic, and neural cells in the presence of lineage-specific induction environment [11]. These characteristics endow ADSCs regenerative properties. Therefore, ADSCs are ideal candidates for cell-based therapies in the tissue engineering and regenerative medicine [12]. However, little is known about the expression and function of TIMP-1 in ADSCs. In this study, we investigated whether TIMP-1 can regulate the proliferation of ADSCs. Our findings indicated that TIMP-1 knockdown significantly inhibited cell proliferation and blocked cell cycle progression at G_1_ phase in the ADSCs. The results suggest that TIMP-1 functions as a positive regulator of ADSCs proliferation and may accelerate the application of ADSCs in regenerative medicine.

## 2. Materials and Methods

### 2.1. Isolation and Culture of ADSCs

ADSCs were isolated from the lipoaspirates of healthy human donors as we described previously [13, 14]. The written informed consents were obtained from the donors of adipose tissues. This study was reviewed and approved by the Human Research and Ethical Committee of Affiliated Hospital of Guangdong Medical College. The isolated ADSCs were cultured at 37°C in low glucose DMEM medium (Gibco, USA) added with 10% fetal bovine serum (FBS) in a humidified incubator with 5% CO_2_, and the cells at passages 4–6 were used for the following experiments.

### 2.2. Expression Level of TIMP-1 Protein

Expression levels of TIMP-1 protein in adipose tissue, fibroblasts, and ADSCs were detected by western blotting. Briefly, 20 *μ*g total protein from each sample was separated on a 10% SDS polyacrylamide gel and transferred to polyvinylidene difluoride (PVDF) membranes. Subsequently, the membranes were incubated with primary antibodies of TIMP-1 (Abcam, Cambridge, MA) and *β*-actin (Abcam) at 4°C overnight, followed by incubation with secondary antibody for 2 h at room temperature. The protein bands were then visualized with an enhanced chemiluminescence system (Amersham Biosciences, NJ, USA).

### 2.3. Construction of TIMP-1 shRNA Lentiviral Vector and Infection into Cells

The shRNA sequences were used in the following experiments: TIMP-1 shRNA (shTIMP-1): 5′-GTTGTTGCTGTGGCTGATA-3′; the nonsilencing shRNA (mock): 5′-TTCTCCGAACGTGTCACGT-3′. They were, respectively, cloned into the GV248 vector (GeneChem, Guangzhou, China) that contained GFP and puromycin resistance gene. The recombinant virus was packaged in 293T cells using a lentivector expression system (GeneChem) according to the manufacturer's instructions, followed by cellular infection. ADSCs were infected with lentivirus-mediated shRNA-TIMP-1 or mock-shRNA when they had grown to about 80% confluence. The GFP expression level was observed using fluorescence microscopy to determine the infection efficiency. The infected ADSCs were subcultured in puromycin-containing growth medium for 3 days to detect the expression level of TIMP-1 with real-time RT-PCR and western blotting, respectively.

### 2.4. Quantitative Real-Time RT-PCR

Total RNA was extracted from the ADSCs using TRIzol reagent (Life Technologies, Carlsbad, CA) according to the manufacturer's instructions. The concentration of total RNA was determined by measuring the absorbance at 260 nm. 2 *μ*L total RNA was reverse-transcribed into cDNA using oligo(dT)_20_ primer and MML-V reverse transcriptase (Takara, Japan), followed by real-time PCR with the reagent SYBR Premix ExTaq*™* (Takara, Japan) on Light Cycler 480® real-time PCR system. The PCR primers were used as previously reported [15] and are listed as follows: TIMP-1 forward, 5′-ACTTCCACAGGTCCCACAAC-3′; TIMP-1 reverse, 5′-GCATTCCTCACAGCC AACAG-3′; GAPDH forward, 5′-TGCACCACCAACTGCT TAG-3′; GAPDH reverse, 5′-GTTCAGCTCAGGGATGACC-3′. PCR amplification was conducted at 95°C for 3 min, 45 cycles at 95°C for 30 sec, 60°C for 45 sec, followed by 72°C for 5 min. The relative expression level of TIMP-1 mRNA was calculated by normalization to the GAPDH mRNA level.

### 2.5. Cell Proliferation Assay

Cell proliferation was evaluated by MTT assay. Briefly, the cells were plated in 96-well plates at a density of 5 × 10^3^ cells/well and incubated at 37°C for 24, 48, 72, and 96 h after infection, followed by addition of 10 *μ*L MTT solution (5 mg/mL) to each well for 4 h. The absorbance was recorded at 570 nm with automated spectrophotometric plate reader (PerkinElmer, USA). The experiments were repeated at least three times.

### 2.6. Immunocytochemical Staining

Immunocytochemical staining for the proliferative marker, Ki67, was performed as described previously [14], with some modifications. Briefly, the cells were seeded on the glass slides at a density of 5 × 10^3^ cells per slide 72 h after the infection with lentivirus-mediated shRNA-TIMP-1 or mock-shRNA. After 24 h of incubation, the slides were washed twice with PBS and fixed in 4% paraformaldehyde at room temperature, followed by incubation with a monoclonal Ki67 antibody (1 : 400; ZSGB-BIO, Beijing, China) for 12 h at 4°C. After washing, biotinylated anti-rabbit IgG secondary antibody was added for 20 min of incubation, followed by incubation with avidin-biotin-peroxidase complex (Vector Laboratories Ltd., Peterborough, UK) for 1 h. Negative control was made without the primary antibody. The slides were evaluated at 400x magnification under the microscope with a digital camera. The percentage of positive Ki67 staining was quantified by ratio of Ki67-positive cells in four different visual areas to total cell number in corresponding areas in each sample.

### 2.7. Cell Cycle Analysis

Cell were harvested, washed twice with PBS, and fixed in 75% ethanol for 1 h. The cells were then centrifuged to remove the 75% ethanol and incubated in cell cycle staining solution containing 50 *μ*g/mL of propidium iodide (Beyotime, China) for 30 min in the dark at room temperature. The percentage of cells at different phases of cell cycle was determined by flow cytometry (Becton Dickinson, NJ, USA).

### 2.8. Western Blotting

Protein expression levels of p53, p21, cyclin E, CDK2, and P-CDK2 (CST Company, Danvers, USA) in the parental ADSCs, mock-shRNA ADSCs, and shTIMP-1 ADSCs were determined by western blotting according to standard procedures. The intensities of protein bands were quantified by Bio-Rad Quantity One software (Bio-Rad, USA). *β*-actin was used as a loading control.

### 2.9. Statistical Analyses

All values were expressed as mean ± standard deviation (SD) and analyzed using a SPSS 13.0 statistical package. The comparison between means of two groups was assessed by unpaired Student's *t*-tests. *P* < 0.05 was considered statistically different.

## 3. Results

### 3.1. Expression Level of TIMP-1 Protein in ADSCs

**Figure 1:**
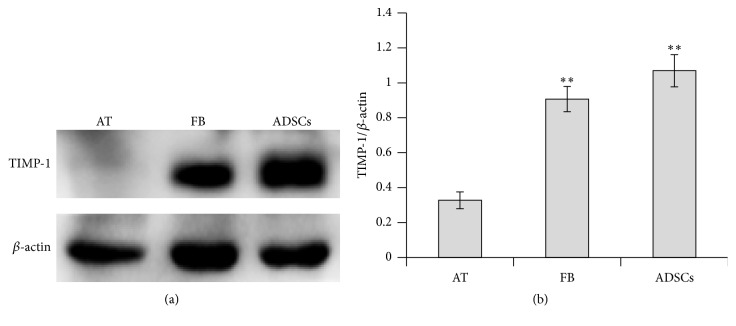
Western blot analysis for TIMP-1 protein expression. The expression levels of TIMP-1 protein in adipose tissue, fibroblasts, and ADSCs were determined by western blotting analysis. (a) The representative western blot images for the bands of TIMP-1. (b) Relative expression levels of TIMP-1 protein were quantified by the densitometry of each band normalized to *β*-actin signal. The data represent the mean ± SD (*n* = 3). ^*∗∗*^ *P* < 0.01 versus the AT group. AT: adipose tissue; FB: fibroblasts.

To investigate the possible role of TIMP-1 in ADSCs, we firstly analyzed the levels of TIMP-1 protein expressed in the adipose tissues, fibroblasts, and ADSCs by western blotting. As shown in Figure 1, expression level of TIMP-1 protein was considerably higher in fibroblasts and ADSCs than in the adipose tissue.

### 3.2. TIMP-1 Knockdown and Its Effects on Cell Proliferation

**Figure 2:**
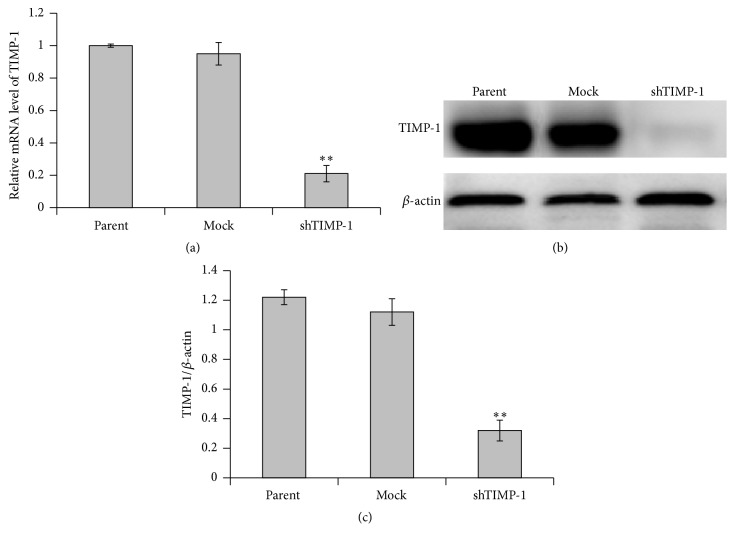
TIMP-1 expression was suppressed by shRNA-TIMP-1. TIMP-1 expression was analyzed by real-time RT-PCR and western blotting, respectively. (a) The levels of TIMP-1 mRNA in parental ADSCs, mock, and shTIMP-1 ADSCs were measured by real-time RT-PCR 72 h after shRNA infection. TIMP-1 mRNA expression in parental ADSCs is defined as 1. The *y*-axis represents the normalized TIMP-1 mRNA expression relative to parental ADSCs. (b) Representative western blot images for the detection of TIMP-1 protein expression in the ADSCs infected with shRNA against TIMP-1. (c) The relative expression level of TIMP-1 protein was quantified by the ratio of optical density of TIMP-1 and *β*-actin. The data are denoted as mean ± SD (*n* = 3). ^*∗∗*^ *P* < 0.01 versus mock-shRNA group. Parental ADSCs: uninfected ADSCs; mock ADSCS: control lentivirus infected ADSCs; shTIMP-1 ADSCs: shRNA-TIMP-1 lentivirus infected ADSCs.

To elucidate whether TIMP-1 expression has any effect on the proliferation of ADSCs, RNAi was used to generate TIMP-1 knockdown cell lines. In this study, lentivirus-mediated infection was performed in ADSCs. The results of RT-PCR confirmed that TIMP-1 mRNA expression in the shTIMP-1 ADSCs was suppressed by about 80% compared to the cells infected with mock-shRNA (Figure 2(a)). Additionally, western blot analysis demonstrated that TIMP-1 protein expressed in the shTIMP-1 ADSCs was also markedly downregulated (Figures 2(b) and 2(c)), whereas TIMP-1 protein bands were clearly observed in the mock-shRNA cells and parental ADSCs.

**Figure 3:**
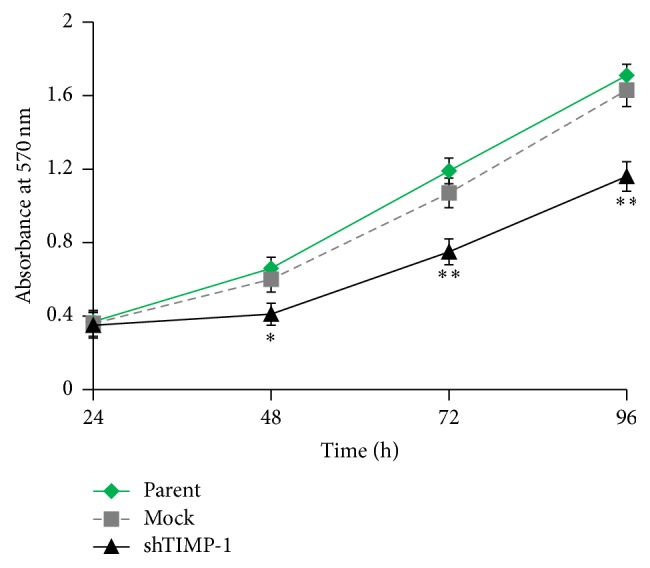
siRNA-mediated downregulation of TIMP-1 inhibits the proliferation of ADSCs. Cell growth curves during 96 h were evaluated by MTT assay. Data are the mean absorbance values of three measurements and the bars represented SD of the mean. ^*∗*^ *P* < 0.05 and ^*∗∗*^ *P* < 0.01 versus mock-shRNA groups. Parent: uninfected ADSCs; mock: control lentivirus infected ADSCs; shTIMP-1: shRNA-TIMP-1 lentivirus infected ADSCs.

**Figure 4:**
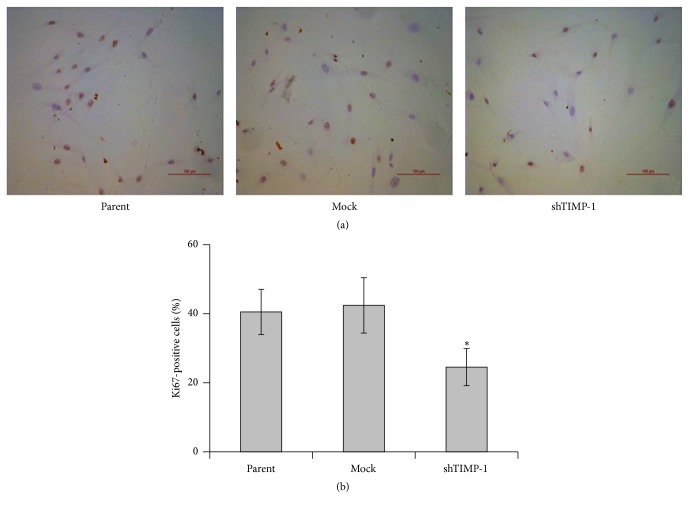
Immunocytochemical staining of Ki67. Ki67 staining was performed to evaluate cell proliferation after TIMP-1 knockdown in the ADSCs. (a) Representative images of Ki67 staining in the parental ADSCs, mock-shRNA ADSCs, and shTIMP-1 ADSCs. (b) Percentage of Ki67 positive cells was quantified in four random areas as described in Section 2. The data are denoted as mean ± SD (*n* = 4). ^*∗*^ *P* < 0.05 versus mock-shRNA groups. Scale bar: 100 *μ*m.

On the other hand, MTT assay was performed to evaluate the effect of TIMP-1 knockdown on cell proliferation. Decreased cell proliferation was observed in the shTIMP-1 ADSCs relative to mock-shRNA cells and parental ADSCs (Figure 3), which suggest that TIMP-1 knockdown strongly inhibits the proliferation and growth of ADSCs. To further confirm the inhibitory effect of TIMP-1 knockdown on the proliferation of ADSCs, Ki67 staining was performed. As shown in Figure 4, the percentage of Ki67 positive cells (brownish) was significantly reduced in the shTIMP-1 ADSCs compared with the mock-shRNA control cells or parental ADSCs.

### 3.3. Effect of TIMP-1 Knockdown on Cell Cycle Progression

**Figure 5:**
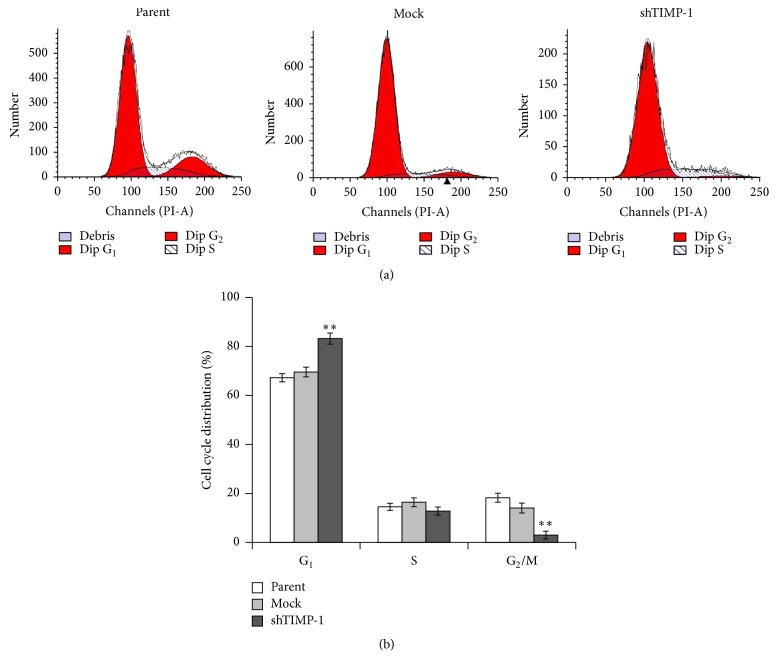
TIMP-1 knockdown blocked cell cycle progression. Cells were stained with propidium iodide and cell cycle distribution was then analyzed using flow cytometry 72 h after infection. (a) Representative images of cell cycle distribution in parental, mock-shRNA, and shTIMP-1 ADSCs. (b) The histogram was the statistical data from three independent experimental replicates. ^*∗∗*^ *P* < 0.01 versus the mock-shRNA groups.

To investigate the effect of TIMP-1 knockdown on cell cycle progression, cell cycle distribution of parental ADSCs, mock-shRNA ADSCs, and shTIMP-1 ADSCs was analyzed by flow cytometry. As shown in Figure 5, a significantly increased percentage of cells at G_1_ phase was observed in the shTIMP-1 cells along with a marked decline in the population of G_2_/M phase cells, which suggests that TIMP-1 knockdown strongly blocked cell cycle progression of ADSCs.

**Figure 6:**
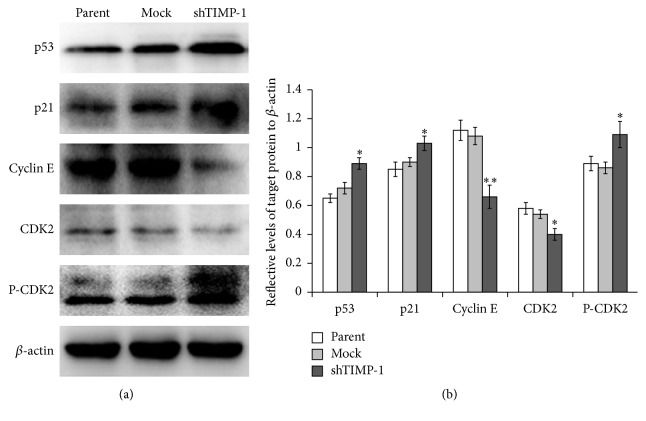
Effects of TIMP-1 knockdown on p53 protein and cell cycle biomarkers. The levels of p53, p21, cyclin E, CDK2, and P-CDK2 proteins were analyzed by western blotting after TIMP-1 knockdown. (a) The representative western blot images. (b) The expression levels of p53, p21, cyclin E, CDK2, and P-CDK2 were quantified by the densitometry of each band and were normalized by internal loading control, *β*-actin. The data represent the mean ± SD (*n* = 3). ^*∗*^ *P* < 0.05 and ^*∗∗*^ *P* < 0.01 versus the mock-shRNA groups. Parent: uninfected ADSCs; mock: control lentivirus infected ADSCs; shTIMP-1: shRNA-TIMP-1 lentivirus infected ADSCs.

It is well-known that p53 gene regulates the expression and activity of downstream genes that control cell cycle progression [16]. The levels of p53 and its downstream gene p21 proteins expressed in shTIMP-1 ADSCs were significantly upregulated compared with the control ADSCs (Figure 6). Additionally, the expression level of cyclin E, a relevant cyclin regulating G_1_/S progression, was found to be downregulated after TIMP-1 knockdown in the ADSCs. CDK2 plays a critical role in controlling the progression of cell cycle from G_1_ to S phase [17]. The results of western blotting demonstrated that the level of CDK2 was decreased, but its phosphorylated form was upregulated in the ADSCs after TIMP-1 knockdown.

## 4. Discussion

In this study, we first investigated the expression level of TIMP-1 in ADSCs and found that the constitutive high expression of TIMP-1 was observed in the ADSCs and fibroblasts, and its expression level was very limited in the human adipose tissue. The downregulation of TIMP-1 expression induced by specific shRNA in the ADSCs significantly inhibited cell proliferation and arrested cell cycle progression possibly through the regulation of cell cycle-associated protein expression. These findings suggest that TIMP-1 expression promotes cell growth and cell cycle progression of ADSCs.

Since TIMP-1 was discovered about two decades ago as a MMP inhibitor, it has been found to be expressed in various cell types such as fibroblasts, osteoblasts, epithelial and endothelial cells, smooth muscle cells, chondrocytes, mesenchymal stem cells, and many tumor cells [9, 15, 18]. TIMP-1 expression plays various roles in the regulation of cell growth and apoptosis [3]. It was reported that TIMP-1 expression promoted epithelial cell proliferation during mouse mammary development [19]. But other studies demonstrated that TIMP-1 reduced the growth rate of human breast epithelial cells (MCF10A) by inducing cell cycle arrest at G_1_ phase [20]. These conflicting data suggest that TIMP-1 expression results in different effects on cell growth in various cell types. The specific effects of the TIMPs on cell fate probably depend on the cell context and specific model system under study [4].

In the present study, TIMP-1 expression was successfully downregulated in the ADSCs through lentivirus-mediated shRNA against TIMP-1. MTT assay and Ki67 staining both showed that cell proliferation was significantly suppressed in the shTIMP-1 ADSCs, which was possibly associated with cell cycle arrest. The results of flow cytometry analysis confirmed cell cycle arrest at G_1_ phase in the ADSCs after TIMP-1 knockdown. It is well-known that p53 plays an important role in the regulation of cell cycle arrest, senescence, cell metabolism, apoptosis, or autophagy [21]. In this study, the expression levels of p53 and p21 proteins were both significantly upregulated when TIMP-1 was silenced in the ADSCs. p21 is one of the downstream genes of p53, and it has been found to modulate cell cycle progression at G_1_/S phase transition [22]. Thus, our results indicated that TIMP-1 knockdown resulted in cell cycle arrest at G_1_ phase and reduced the percentage of S and G_2_/M phase cells possibly through the modulation of p53-p21 signaling pathway. This is similar to previous reports that TIMP-1 reduced the growth rate of the hepatocellular carcinoma cells (BEL-7402) by upregulation of p21 [23]. Additionally, p21 is a negative regulator of cyclin-dependent kinase (CDK) inhibitor and can interact with CDK including CDK2 complexes that control the G_1_/S phase transition resulting in cell cycle arrest [24, 25]. Therefore, we detected the expression levels of cyclin E and CDK2 proteins in the ADSCs after TIMP-1 knockdown. The results indicated that shRNA-mediated downregulation of TIMP-1 significantly reduced the expression levels of cyclin E and CDK2 proteins but increased the production of CDK2 phosphorylated forms in the shTIMP-1 ADSCs, which at least partly contributed to cell cycle arrest in these cells.

In conclusion, we have demonstrated that TIMP-1 downregulation by shRNA against TIMP-1 gene significantly inhibited cell proliferation and induced cell cycle arrest at G_1_ phase in the ADSCs possibly through upregulating the expression levels of p53, p21, and P-CDK2 and reducing the production of cyclin E and CDK2 proteins. These findings suggest that TIMP-1 is a positive regulator of cell growth in human ADSCs.
